# Perceptions and Discussions of Snus on Twitter: Observational Study

**DOI:** 10.2196/38174

**Published:** 2022-08-29

**Authors:** Jiarui Chen, Siyu Xue, Zidian Xie, Dongmei Li

**Affiliations:** 1 Goergen Institute for Data Science University of Rochester Rochester, NY United States; 2 Department of Clinical & Translational Research University of Rochester Medical Center Rochester, NY United States

**Keywords:** snus, Twitter, sentiment, topic modeling, smokeless tobacco products

## Abstract

**Background:**

With the increasing popularity of snus, it is essential to understand the public perception of this oral tobacco product. Twitter—a popular social media platform that is being used to share personal experiences and opinions—provides an ideal data source for studying the public perception of snus.

**Objective:**

This study aims to examine public perceptions and discussions of snus on Twitter.

**Methods:**

Twitter posts (tweets) about snus were collected through the Twitter streaming application programming interface from March 11, 2021, to February 26, 2022. A temporal analysis was conducted to examine the change in number of snus-related tweets over time. A sentiment analysis was conducted to examine the sentiments of snus-related tweets. Topic modeling was applied to tweets to determine popular topics. Finally, a keyword search and hand-coding were used to understand the health symptoms mentioned in snus-related tweets.

**Results:**

The sentiment analysis showed that the proportion of snus-related tweets with a positive sentiment was significantly higher than the proportion of negative sentiment tweets (4341/11,631, 37.32% vs 3094/11,631, 26.60%; *P*<.001). The topic modeling analysis revealed that positive tweets focused on snus’s harm reduction and snus use being an alternative to smoking, while negative tweets focused on health concerns related to snus. Mouth and respiratory symptoms were the most mentioned health symptoms in snus-related tweets.

**Conclusions:**

This study examined the public perception of snus and popular snus-related topics discussed on Twitter, thus providing a guide for policy makers with regard to the future formulation and adjustment of tobacco regulation policies.

## Introduction

Smokeless tobacco is a type of tobacco that is neither smoked nor burnt during consumption. Examples of smokeless tobacco products include chewing tobacco, dissolvable tobacco, and oral nicotine pouches. According to the Centers for Disease Control and Prevention (CDC), in 2020, there were 5.7 million adult users of smokeless tobacco nationwide in the United States [1]. Among the smokeless tobacco products, snus is a smokeless and sometimes flavored tobacco product for oral consumption that originated from Sweden. It is usually in the following two forms: loose ground powder and sachets. When snus is consumed, it is held behind the upper lip [2]. Although this tobacco product was banned in the member countries of the European Union, with a few exceptions such as Sweden [3], its use in the rest of the world is prevalent. By 2013 for example, 18% of adolescents had tried snus in Finland [4]. In the United States, a study conducted in 2021 by the CDC suggested that 1.2% of US high school students are current users of smokeless products, including snus [1].

Studies have found that snus use may result in oral cancer, cardiovascular diseases, respiratory diseases, diabetes, and other illnesses [5]. A cohort study on 135,036 male, Swedish construction industry employees found that the age-adjusted relative risk of dying from cardiovascular disease for smokeless tobacco users was 40% higher than that for nonusers [6]. Despite these concerns, previous studies indicated that snus use has a considerably lower health risk than cigarette smoking [2,7]. According to a review on multiple health symptoms, including oral health and cardiovascular diseases, among others, the health risk of snus is significantly lower than that of cigarettes [2].

Similar to other tobacco products, snus use results in nicotine dependence, and the perceptions toward the relationship between snus consumption and other types of nicotine consumption have been controversial [5]. The gateway hypothesis states that the use of snus may lead to more addictive smoking behaviors. On the contrary, the pathway hypothesis claims that snus use helps to prevent people from smoking [5]. Previous studies on this topic reported different conclusions. A previous study tracked 496 pairs of users and nonusers of smokeless tobacco products and concluded that there was insufficient evidence to conclude that using smokeless tobacco products leads to a higher chance of smoking [8]. Another research study on smokers in Sweden found that 76.3% of the male smokers and 71.6% of the female smokers included in the study quit smoking after they started consuming snus [9]. However, a focus group study that was performed on 66 participants in 2010 concluded that the participants believed that snus use could potentially lead to smoking [10].

With the controversial gateway and pathway hypotheses and the potential health impact of snus products, disagreements on the perception of snus product may exist among the public. As snus is becoming increasingly popular, governmental regulation plays an essential role in the relationship between snus consumption and public health. For example, the US Food and Drug Administration stipulates that for smokeless tobacco products, including snus, special warnings such as “WARNING: Smokeless tobacco is addictive” should be attached to the packages [11]. For governors and regulators to better manage the relationship between snus and public health and be more informed in policy making, it is beneficial to understand how the public truly perceives snus.

Twitter, as a popular social media platform, has been used to examine smoking behaviors and perceptions of tobacco products, such as e-cigarettes [12,13]. Although perceptions of snus have been investigated by using focus groups, the sample sizes of such focus groups are very limited [10,14]. Research that uses social media data to study the public perceptions of snus is scarce.

Our study aimed to examine the public perceptions of and popular topics regarding snus on Twitter. Our study consisted of 3 specific goals. First, we aimed to determine the sentiments of snus-related tweets via a sentiment analysis. Second, we attempted to explore specific topics related to snus. Finally, we tried to examine potential health risks that were mentioned in snus-related tweets. Through a comprehensive examination of the public perceptions and the top topics discussed about snus, we hope to provide some insights to policy makers on regulating snus for public health protection.

## Methods

### Ethics Approval

We only used publicly available tweets for this study, and there was no identifying information on Twitter users in this study. In addition, this study was reviewed and approved by the Office for Human Subject Protection Research Subjects Review at the University of Rochester (study ID: STUDY00006570).

### Data Collection and Preprocessing

We collected Twitter posts (tweets) related to snus from March 11, 2021, to February 26, 2022, through the Twitter streaming application programming interface by using the keyword *snus*, and we obtained a data set with 28,427 tweets. We then preprocessed the data to enhance their quality. First, all the tweets were lowercased. Afterward, by using the Regular Expression Operations Package (Python Software Foundation) [15], we removed the parts of tweets that did not contribute to the tweets’ actual contents, including email addresses, new-line characters, single quotation marks, URLs, and “@” signs (used to mention other users). Next, we applied 2 sets of promotion filters to eliminate tweets that were related to the commercial promotion of snus [13]. The first filter targeted the usernames, using keywords such as *snus*, *smokeless*, *dealer*, *supply*, *nicotine*, *cigarette*, and *store*. Tweets posted by users with usernames containing any of these words were not included in this study because they might have been posted by commercial accounts. The second layer of the filter aimed to remove potentially commercial tweet content, and the keywords included *order*, *new*, *offer*, *discount*, and *free shipping*. Tweets that contained these words were highly likely to be promotional tweets. Finally, we eliminated the repetitive tweets. After preprocessing, the final data set contained 11,631 tweets.

### Sentiment Analysis

Sentiment analysis is a computational method of learning the attitudes in text, and the Valence Aware Dictionary and Sentiment Reasoner (VADER) is a sentiment analysis package that is specialized for social media data [16]. By applying the VADER on each tweet, we assigned each tweet a sentiment score of between −1.0 and 1.0. To better define the sentiments, we grouped the tweets into 3 categories based on the corresponding sentiment scores; tweets with a sentiment score of ≥0.05 were labeled as “positive,” and tweets with a score of ≤−0.05 were labeled as “negative.” The remaining tweets were labeled as “neutral.” The proportions of positive, neutral, and negative tweets were then calculated. The daily proportion of positive tweets was then calculated.

We performed the chi-square goodness-of-fit test by using statistical analysis software (R version 4.0.2; R Foundation for Statistical Computing) to examine the frequency distribution of different attitudes [17]. A significance level of .05 was used to determine whether the proportion of positive tweets was statistically significantly higher than the proportion of the negative tweets.

### Topic Modeling

Topic modeling is a computational method of identifying major topics in text. The model we chose for our study was the latent Dirichlet allocation model, which was applied to positive tweets, neutral tweets, and negative tweets to observe the main topics that Twitter users had been discussing.

By using the *gensim* package in Python [18], we built a bigram and trigram based on our data set. Bigrams and trigrams are sequences of 2 words and 3 words, respectively. With the bigram and the trigram, we treated some of the most frequently mentioned phrases as a whole instead of 2 or 3 separate words. For example, *harm reduction* was a frequently mentioned phrase among the tweets, and we considered *harm reduction* as a single token that contributed to a topic instead of preserving *harm* and *reduction* separately.

We applied the Natural Language Toolkit to remove the stop words in the tweets [19]. Stop words include but are not limited to commonly used articles, pronouns, and propositions, which undermine the quality of topic modeling results if kept. In addition, we used spaCy (Explore) to lemmatize the words in tweets into their dictionary forms without changing their meaning [20]. For example, *smoked* became *smoke* after lemmatization. After conversion, words like *smoked* were left unused for topic modeling, and only their dictionary forms were included. Both coherence scores and intertopic distance maps were used to determine the optimal number of topics discussed in the tweets, using the *pyLDAvis* package in Python [21].

To better interpret the results from the model, we inferred the topics based on the keyword outputs and example tweets. Two authors reviewed the tweets from each category and summarized the topics independently. The results from the two authors were compared and discussed. Any discrepancy was resolved by a group of 4 members.

### Health-Related Discussion

To determine the frequency of health effects that were mentioned in snus-related tweets, we filtered the data set by using a list of health-related keywords that were created in previous studies [22-24], which resulted in a set of 654 unique tweets with 1254 health-related keyword appearances. The list included the following nine major groups of health effects that are related to smoking and nicotine consumption: mouth (eg, gum, teeth, etc), respiratory (eg, lung, cough, etc), cardiovascular (eg, heart, etc), psychological (eg, stress, anxiety, etc), neurological (eg, numb, fatigue, etc), cancer (eg, lung cancer, mouth cancer, etc), throat, digestive, and other effects (eg, skin, liver, etc). For each major group of health effects, the number of occurrences of specific keywords belonging to the groups were counted. In addition, two authors hand-coded 200 randomly selected tweets to determine whether the users directly experienced the health symptoms mentioned or whether they believed that snus use might help with lowering the risk of the symptoms when compared to smoking. The Cohen κ statistic reached 0.73, indicating substantial agreement between the two coders.

## Results

### Temporal Analysis

**Figure 1:**
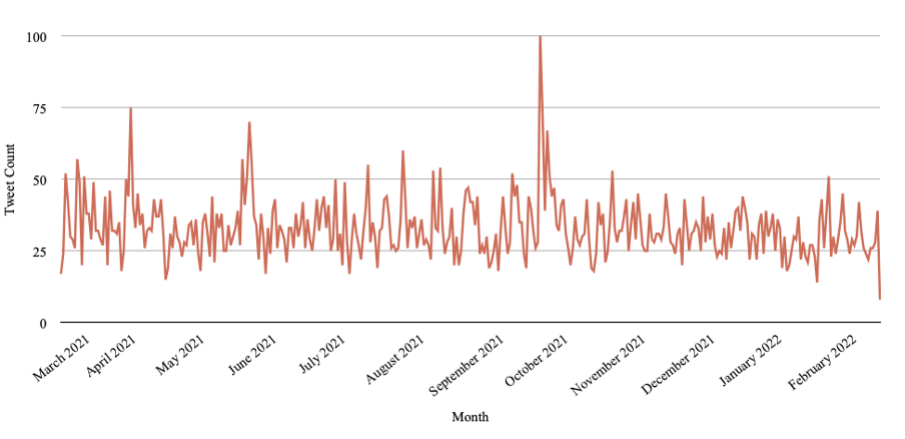
Snus-related tweets from March 11, 2021, to February 26, 2022.

To better understand the popularity of snus discussion, we examined the number of snus-related tweets over time during our study time period. As shown in Figure 1, the number of tweets per day typically oscillated between 25 and 50, with a few peaks occurring in April 10, 2021; May 31, 2021; and October 3, 2021.

### Perceptions of Snus on Twitter

To examine the public perception of snus on Twitter, we performed a sentiment analysis on tweets related to snus. The average sentiment score for 11,631 snus-related tweets was 0.080, which indicated that the overall sentiment in snus-related tweets was positive. Among these tweets, there were 4341 (37.32%) positive tweets, 3094 (26.60%) negative tweets, and 4196 (36.08%) neutral tweets. Further statistical analysis showed that the proportion of positive tweets was significantly higher than the proportion of negative tweets (4341/11,631, 37.32% vs 3094/11,631, 26.60%; *P*<.001). Our longitudinal analysis showed that there was no noticeable change in the proportion of positive tweets over time (Multimedia Appendix 1).

### Topics Discussed in Snus-Related Tweets

To understand what might be responsible for different sentiments in snus-related tweets, we performed topic modeling for the tweets in the different sentiment groups. As shown in Table 1, among the positive sentiment snus-related tweets, the most popular topic was “Snus being a safer way of nicotine consumption” (1472/4341, 33.9%), followed by “Way of snus consumption” (1441/4341, 33.2%) and “Snus addiction and enjoyment” (1428/4341, 32.9%). Among the negative sentiment snus-related tweets, the top topic was “Risk comparison between snus and smoking” (1064/3094, 34.4%), followed by “Negative health impacts” (1018/3094, 32.9%) and “Other problems related to snus” (1012/3094, 32.7%). The topics for neutral sentiment snus-related tweets are included in Table S1 in Multimedia Appendix 2.

**Table 1:** Topics discussed in snus-related tweets with different sentiments.

Sentiment group and inferred topic			Keywords		Token percentage		Examples
**Positive**							
	Snus addiction and enjoyment	*snus*, *good*, *make*, *time*, *day*, *love*, *feel*, *free*, *access*, *today*, *strong*, *man*, *back*, *coffee*, *pack*, *life*, *pretty*, *friend*, *enjoy*, and *week*		32.9		“Proper pint of bitter and a wintergreen snus. Perfect on a fair night like tonight”	
	Snus being a safer way of nicotine consumption	*pouch*, *vape*, *smoking*, *smoke*, *quit*, *cigarette*, *nicotine*, *safe*, *give*, *amp*, *year*, *alternative*, *smoker*, *start*, *risk*, *big*, *stop*, *switch*, *low*, and *option*		33.9		“For long-term nicotine use, data on safety are strongest for snus: decades of epidemiological studies. No harm. So if many people with mental health issues self-medicate with #safernicotine (they are), at least there is no harm. #qualityoflife”	
	Way of snus consumption	*snus*, *tobacco*, *product*, *Swedish*, *people*, *chew*, *work*, *thing*, *great*, *smokeless*, *put*, *dip*, *find*, *call*, *play*, *gum*, *nice*, *hard*, *flavor*, and *mouth*		33.2		“snus is a black tobacco product you chew or put on your gums. You don’t snort it or sniff it. He’s clearly closing one nostril to sniff smelling salts, which are commonly used in sports. Not rocket science.”	
**Negative**							
	Risk comparison between snus and smoking	*Tobacco*, *smoke*, *vape*, *cigarette*, *smoking*, *pouch*, *product*, *cancer*, *risk*, *low*, *nicotine*, *amp*, *quit*, *harm*, *gum*, *rate*, *smoker*, *chew*, *reduce*, and *smokeless*		34.4		“not just snus but the attempt to restrict and eliminate all lower risk products is astonishingly short sighted.”	
	Negative health impacts	*Snus*, *ban*, *make*, *stop*, *day*, *Swedish*, *year*, *give*, *feel*, *thing*, *death*, *man*, *start*, *high*, *mouth*, *kill*, *lose*, *addiction*, *long*, and *cig*		32.9		“not in epok which i assume is some zoomer snus? i dont know i only use odens and sometimes siberia which has no flavouring just tobacco. the nicotine content is pretty potent in those, would kill your average vaper no joke.”	
	Other problems related to snus	*snus*, *people*, *time*, *bad*, *work*, *put*, *hard*, *good*, *study*, *week*, *today*, *back*, *call*, *big*, *find*, *coffee*, *problem*, and *life*		32.7		“our big daddy is always the leader he is the familys captain and chief, but once i choked when my snus caught up in my throat cause there was our pop in the oak.”	

### Health Risks Mentioned in Snus-Related Tweets

To understand what health risks might be associated with snus, we explored the health symptoms mentioned in the snus-related tweets. Oral health (mouth effects) was the most mentioned health category in snus-related tweets (519/1254, 41.39%), followed by other effects (213/1254, 16.99%) and respiratory effects (182/1254, 14.51%). The other health categories had relatively lower proportions of tweets. For example, the cancer category (cancer is a health effect that is often associated with nicotine consumption) only took up 5.34% (67/1254) of the total tweets. Further hand-coding results showed that of the 200 randomly selected tweets, 40 (20%) mentioned that the health symptoms were a direct result of snus consumption or mentioned a negative opinion about snus. In addition, 28% (56/200) of the tweets discussed the harm reduction of snus, in terms of the health symptoms mentioned, when compared to smoking.

## Discussion

### Principal Findings

In our study, we showed that the proportion of snus-related tweets with a positive sentiment was significantly higher (*P*<.001) than the proportion of snus-related tweets with a negative sentiment. By using topic modeling, we observed that the positive sentiments toward snus might be the result of personal experiences and the perception that snus use is a safer alternative to smoking. In contrast, concerns about health risks might contribute to the negative sentiments in snus-related tweets. A further analysis showed that in snus-related tweets, the most popular health category was mouth effects, followed by other effects (eg, liver and skin effects) and respiratory effects.

### Comparison With Previous Studies

Our temporal analysis showed an obvious peak in the number of snus-related tweets on October 3, 2021. After extracting all snus-related tweets from that day, we noticed that most of the tweets (67/100, 67%) discussed the possible use of snus by the son of a famous English former soccer player. This peak indicates the large impact of influencers on Twitter users.

Given that the top topic in snus-related tweets with a positive sentiment was related to switching from smoking to snus use, since snus was perceived as a safer option and there was no strong evidence in negative sentiment tweets indicating the gateway effect, it might be possible that Twitter users’ perceptions on snus tend to lean toward the pathway hypothesis instead of the gateway hypothesis. This finding contradicts that of a focus group study, in which participants viewed snus use as a potential gateway to smoking [10]. There are 2 possible reasons for this inconsistency. First, the focus group was conducted in 2010, and the tweets used in our study were collected in 2021. It is possible that temporal differences might account for the difference in the perceptions of snus. Second, the conclusion from the focus group was based on a sample of 66 young adults who ranged in age from 18 to 26 years [10]. In comparison, our study included a broader range in terms of demographic characteristics, which may have led to the different results.

From the aspect of health risks, the health-related keywords identified in the tweets captured the majority of the potential health impact of snus. According to a report published by the Norwegian Institute of Public Health in 2019, the main potential adverse health effects of snus cover cancer, cardiovascular disease, mental disorders, and caries [25]. The health-related keyword frequency distribution from our study included these potential health effects through the oral, cardiovascular, cancer, and psychological effect categories, demonstrating the consistency between our findings from Twitter data and previous findings on the health risks of snus.

### Limitations

Our study has several limitations. Data collected from Twitter may contain some bias. A study on tourist attraction visit sentiment data sourced from Twitter suggested that the tourists’ sentiments could be affected by factors other than the tourist attraction itself, including the number of attraction sites that are visited in 1 day and whether the tourists are local visitors, out-of-state visitors, or international visitors [26]. Another study in 2012 suggested that the demographic distributions of Twitter users are different from those of the general population [27]. For example, around 31% of young adults who ranged in age from 18 to 24 years used Twitter, while this proportion was only 17% for adults aged between 25 and 34 years [27]. Therefore, our findings, which are based on Twitter data, may not represent the general population.

With regard to data collection and preprocessing, the keyword set we used may not have been comprehensive. For example, when collecting the data, we only included *snus* as the single keyword, which may have resulted in us missing some relevant tweets in our study. Additionally, in the processed data set, there might have still been some bot accounts, which can automatically deliver messages. This may have introduced some bias in our results. With regard to topic modeling, inferences based on keywords involve subjective judgments, even with the support of example tweets. In addition, the mentioning of health symptoms in snus-related tweets does not imply any causal relationship between snus and health risks. Our hand-coding results further validated this notion. Moreover, our study did not include the demographic information of Twitter users. Different demographic groups might perceive snus differently.

### Conclusion

Our study showed more positive sentiments in snus-related tweets from Twitter users, which might have been due to the relative safety of snus when compared to that of smoking. Our study provided an efficient measurement of the public perceptions of snus among a relatively large sample by using social media data. According to the health belief model, the perceived susceptibility, seriousness, benefits, and barriers of actions explain health-related behaviors [28]. Therefore, these perceptions of snus are possibly a predictor of the public’s snus consumption patterns. Our study will help policy makers better anticipate consumption behavior changes and make necessary policy changes. The results from our study will provide insights to policy makers on further regulations for snus. Future studies could take demographic and geographic factors into consideration to explore potential disparities in snus-related perceptions and discussions.

## Appendix

Multimedia Appendix 1 Proportion of snus-related positive tweets over time.

Multimedia Appendix 2 Table S1. Topics mentioned in snus-related tweets with a neutral sentiment.

## Abbreviations

CDC: Centers for Disease Control and Prevention
FDA: Food and Drug Administration
NIH: National Institutes of Health
VADER: Valence Aware Dictionary and Sentiment Reasoner

## Data Availability

The data and scripts used for analysis and for creating the figures are available on request from the corresponding author (DL).
