# Supplementary figures and images for: Perceptions and Discussions of Snus on Twitter: Observational Study
Source: JMIR Med Inform. 2022 Aug 29;10(8):e38174. doi: 10.2196/38174 (PMC9468913; doi:10.2196/38174)

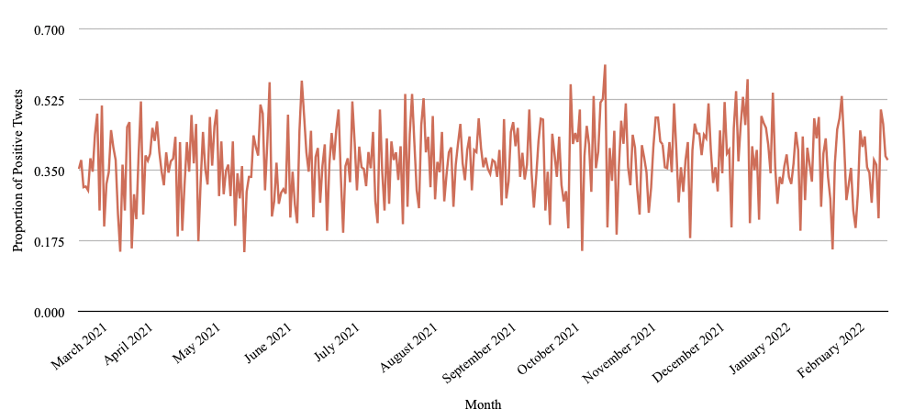

Supplement: Multimedia Appendix 1 [file medinform_v10i8e38174_app1.png]
