# Supplementary material for: Perceptions and Discussions of Snus on Twitter: Observational Study
Source: JMIR Med Inform. 2022 Aug 29;10(8):e38174. doi: 10.2196/38174 (PMC9468913; doi:10.2196/38174)
Supplement: Multimedia Appendix 2 [file medinform_v10i8e38174_app2.docx]

**Table S1.** Topics mentioned in snus-related tweets with a neutral sentiment.

| Sentiment Group | Keywords | Token Percentage | Sample Tweets |
| --- | --- | --- | --- |
| Neutral | tobacco, time, day, make, vape, cigarette, product, put, man, today, chew, thing, nicotine, smoking, call, back, start, mouth, dip, switch | 33.7% | - “he’s not snorting anything. he’s closing one nostril to sniff smelling salts. either that or doing a line of snus which would be a world first for anyone” |
|  | Snus, pouch, Swedish, smoke, quit, pack, work, amp, addict, gum, hit, feel, eat, week, leave, month, long, report, cig, review | 33.3% | - “10 years ago st. patricks day was the day i decided to quit smoking after 27 years of 2 packs a day. it took some time, but i have yet to conquer my addiction to nicotine. switched to mouth tobacco, called snus.” |
|  | Snus, access, give, ratszone, people, year, coffee, bring, find, trade, guy, high, real, night, morning, game, hour, swede, sleep, video | 33% | - “12/ with substance usage, be it needle exchanges, carrying narcan, or reducing alcohol consumption, and reducing the amount of toxic substances introduced to the human body by using substances- vaping, using snus, nicotine replacement therapy, or a combination of those.” |
